# Patient‐derived organoid culture in epithelial ovarian cancers—Techniques, applications, and future perspectives

**DOI:** 10.1002/cam4.6521

**Published:** 2023-09-30

**Authors:** Wai Sun Chan, Xuetang Mo, Philip Pun Ching Ip, Ka Yu Tse

**Affiliations:** ^1^ Department of Obstetrics and Gynaecology The University of Hong Kong Pokfulam Hong Kong SAR; ^2^ Department of Pathology The University of Hong Kong Pokfulam Hong Kong SAR

**Keywords:** epithelial ovarian cancer, patient‐derived organoids, tumor microenvironment

## Abstract

Epithelial ovarian cancer (EOC) is a heterogeneous disease composed of different cell types with different molecular aberrations. Traditional cell lines and mice models cannot recapitulate the human tumor biology and tumor microenvironment (TME). Patient‐derived organoids (PDOs) are freshly derived from patients' tissues and are then cultured with extracellular matrix and conditioned medium. The high concordance of epigenetic, genomic, and proteomic landscapes between the parental tumors and PDOs suggests that PDOs can provide more reliable results in studying cancer biology, allowing high throughput drug screening, and identifying their associated signaling pathways and resistance mechanisms. However, despite having a heterogeneity of cells in PDOs, some cells in TME will be lost during the culture process. Next‐generation organoids have been developed to circumvent some of the limitations. Genetically engineered organoids involving targeted gene editing can facilitate the understanding of tumorigenesis and drug response. Co‐culture systems where PDOs are cultured with different cell components like immune cells can allow research using immunotherapy which is otherwise impossible in conventional cell lines. In this review, the limitations of the traditional in vitro and in vivo assays, the use of PDOs, the challenges including some tips and tricks of PDO generation in EOC, and the future perspectives, will be discussed.

## Graphical abstract

Flat cultures using cell lines and mice models have been used for decades in preclinical cancer research. In recent years, PDOs have revolutionized the field of cancer research as this model can be regarded as a surrogate between mice and human beings and it can recapitulate the tumor features. In this review, the limitations of the traditional in vitro and in vivo assays, the use of PDOs, the challenges including some tips and tricks of PDO generation in EOC, and the future perspectives, will be discussed.

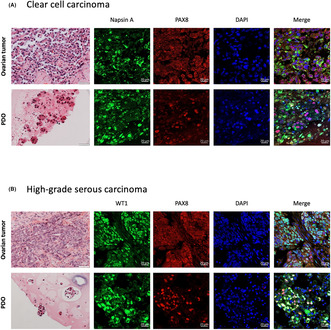

## 1 INTRODUCTION

Over half of patients with advanced epithelial ovarian cancer (EOC) recur within 2 years despite surgery, chemotherapy, and targeted therapy. It is a heterogeneous disease consisting of different histological subtypes. Kurman and Shih suggested a dualistic model to classify EOC.^1^ Type I carcinoma like clear cell carcinoma (CCC) and low‐grade serous carcinoma (LGSC) has stable chromosomes with distinct mutated genes such as *KRAS*, *BRAF*, *PTEN*, and *CTNNB1*, while type II carcinoma like high‐grade serous carcinoma (HGSC), the most common subtype, is characterized by unstable chromosomes, copy number variation (CNV), and *TP53* and *BRCA* mutations.^2^, ^3^, ^4^, ^5^ In addition, different tumors can have different tumor microenvironment (TME), which consists of non‐neoplastic host components including mesenchymal‐derived cells, tissue stroma, fibroblasts, resident or infiltrating vascular structure, and immune cells.^6^ Such tumor heterogeneity highlights the need for personalized medicine.

Traditionally, cell lines, either in two‐dimensional (2D) or spheroid culture, and animals are the cornerstone preclinical models to study cancer biology and evaluate cancer drugs. However, these methods have many limitations. In fact, it is not uncommon that drugs that are proven to be efficacious in preclinical studies fail to demonstrate a benefit in subsequent clinical trials.^7^ These “false‐positive” results may not only consume a lot of resources during drug development but may also lead to unnecessary side effects for the patients. Therefore, a more reliable platform that can recapitulate the human tissues is needed.

Recently, patient‐derived organoids (PDOs) have become an integral part of cancer research. This is because organoids can retain the same genomic features as the primary tumor.^8^ Nevertheless, there are still many pitfalls with PDOs. More complex organoid co‐culture systems incorporating immune and stromal cells have been developed, providing a novel system to study the interaction between different cell types and drugs like immunotherapy. For example, Wan et al. elucidated the mechanism driving the response of BRD1 inhibitor and a bispecific antibody against programmed cell death 1 (PD1) and PD‐ligand 1 (PDL1) in HGSC using organoid co‐culture system.^9^ There are several types of organoid systems, but some of them have not been widely reported in EOC research. In this review, we discuss the limitations of traditional assays, as well as some practical issues, applications, challenges, and future perspectives of PDOs in EOC.

## 2 LIMITATIONS OF CONVENTIONAL MODELS

### 2.1 Cell lines

Cell lines are a simple and inexpensive way to study cancer cell biology and screen for drugs. However, there has been controversy on the putative histology of ovarian cancer cell lines. Indeed, several cell lines that were initially considered as HGSC were found to harbor molecular features of other subtypes instead.^10^, ^11^, ^12^, ^13^ This poses difficulty in selecting the appropriate cell lines that can represent the histological subtypes of interest. In addition, multiple passages of the cell lines may lead to mutations, leading to inconsistent results among different passages. Another major drawback of traditional 2D culture is that the cell lines do not contain TME.^14^ TME controls the plasticity and immunogenicity of the tumor and plays an essential role in tumor carcinogenesis, proliferation, and metastases.^14^, ^15^, ^16^, ^17^ The lack of TME and tumor architecture makes 2D cell line culture difficult to reflect how the cell surfaces interact with each other.

Spheroid using cell lines is a three‐dimensional (3D) in vitro culture model where the multi‐cell spheroids are cultured as free‐floating aggregates. It is a simplified way to simulate the in vivo tumor cell architecture. Due to its nutrient gradient, proliferating cells are mostly concentrated in the outer layer, while quiescent cells are found in the middle layer, and hypoxic and necrotic cells are found in the inner core of the spheroid.^18^, ^19^ The first EOC spheroid model was reported in 1997 to study the resistance of ovarian teratocarcinoma cell spheroids to complement‐mediated killing compared to monolayer culture.^20^ Low‐adhesion environment, supplemented growth factors, extracellular matrix (ECM), tissue factors, fibronectin, collagen, and chondroitin are all favorable factors for spheroid formation.^21^, ^22^ An enriched population of cancer stem cells with stemness markers like CD24, CD44, CD117, and CD133 are often found in ovarian cancer spheroids.^23^ Spheroid formation assay is widely used to investigate TME, cancer stemness, and drug resistance. However, 3D spheroids developed from cell lines do not contain other cell populations like immune cells and mesenchymal cells, and they cannot represent individual patients' genetic variation.

### 2.2 Mice

Animals are used as a surrogate for human tissues and animal studies are required before any products can be tested in human beings. Different animal species such as primates, mice, rats, and zebrafish have been used. Conventional mice models involve inoculation of tumor cell lines and are the most common model as they are relatively simple and inexpensive. Cell lines can also be genetically engineered to study tumorigenesis and drug effects. However, if human cell lines are used, the mice must be immunodeficient thus limiting immune‐oncological research. Murine cell lines can be inoculated in immune‐competent mice to study immune‐oncology. However, these cell lines are not always available, and the mice tissues cannot truly reflect the human immune system. In EOC, the most common commercially available murine cell line is ID8, which was derived from ovarian surface epithelium (OSE) and had wild‐type *Trp53*.^24^

Patient‐derived xenograft (PDX) is another mouse model that involves xenotransplantation of patient‐derived biopsy or body fluid into immunodeficient mice. This method can preserve the TME of human tumors to a certain extent.^25^ Nevertheless, the development of PDX is time‐consuming, labor‐intensive, and expensive. The development of PDX models is also subject to the availability of patients' tumors. The engraftment of the PDX depends on multiple factors, such as the sites and biology of the tumors, previous chemotherapy and surgical treatment, and acquisition methods of the tumors.^26^ For example, there was a challenge in generating low‐grade malignant tumors^27^ while high‐grade tumors tended to have a better uptake rate in mice.^28^ As immunocompromised mice are used, immune‐oncological research is still limited. Furthermore, the difference in the genetic compositions of the recipient mice from the hosts may elicit different responses from human beings.^27^, ^29^

Genetically engineered mouse models (GEMM) and humanized mice can be generated for immune‐related research in oncology. The former involves gene editing to stimulate tumorigenesis which can be done in immune‐competent mice.^30^ Cho's group had developed *Ovgp1‐iCreER* ^*T2*^ mice, where inhibiting *BRCA1*, *Trp53*, *Rb1*, and *Nf1*, *or inhibiting BRCA1*, *Trp53*, and *PTEN*, *in the oviduct led to* serous tubal intraepithelial carcinomas (STICs) and/or HGSC.^30^ They further demonstrated that there was reduced tumor burden in GEMM treated with antibiotics for 12 months compared to the control due to an alteration of the microbiome.^31^ Humanized mice are generated by engrafting human hematopoietic stem cells (HSC) in the mice recipients.^32^ HSCs are most commonly derived from either fetal tissue or cord blood and can develop a functional human‐like immune system in mice.^33^ Gitto et al. transferred patient‐matched tumor‐infiltrating lymphocytes (TILs) to PDX models derived from three HGSC patients and found that the tumor burden was reduced after anti‐PD1 treatment.^34^ However, GEMM and humanized mice cannot provide a fast and high throughput drug screening due to their cost and tedious generation and maintenance procedures. Besides, the physio‐biology and immune composition in mice cannot fully resemble those in humans.^35^, ^36^

## 3 OVERVIEW OF ORGANOID CULTURE

Organoid is another 3D culture model composed of cell aggregates derived from embryonic stem cells, adult stem cells, induced pluripotent stem cells (iPSCs), and/or differentiated cells from human or animal tissues.^37^, ^38^ EOC organoids are tissue‐derived organoids and the starting cell populations commonly include tissue‐resident stem cells, progenitor/differentiated cells, or tumor cells.^39^ Unlike stem cell spheroids generated by commercial stem cell lines or murine‐derived stem cells that preserve the stemness properties and differentiation capacities of stem cell lines only,^40^, ^41^, ^42^, ^43^ organoids preserve some characteristics and functions of the tissues of origin as these are composed of more populations of cells other than stem cells and can recapitulate the genomic landscape of the primary tumor or the tissue of origin.^8^ Both spheroids and organoids are 3D cell aggregates. However, organoids require a scaffolding extracellular environment for self‐assembly, self‐organization, and stem cell differentiation to form a complex structure, and therefore organoids are more physiologically relevant and the tumor structure and TME are better recapitulated compared to spheroids.^44^ The differences between these two models are summarized in Table 1.

**TABLE 1:** Differences between spheroids and organoids.

	Spheroids	Organoids
Source of cells	Cell lines or other primary cells	Human or animal tissues
Cell populations	Tumor cells with and without stem cell properties	Tumor cells, embryonic stem cells, adult stem cells or induced pluripotent stem cells, and other cell populations like immune and mesenchymal cells though these can be lost during the culture
Structural organization	Can be poor as these can be cultured with or without ECM and growth factors	Tumor or organ structures are partially resembled due to the use of ECM and growth factors
TME of the tissue origin	Poorly resembled	Partially resembled
Genomic landscape of the tissue origin	Poorly recapitulated as lack of other cell populations	Partially recapitulated as other cell populations are included

Abbreviations: ECM, extracellular matrix; TME, tumor microenvironment.

Organoid model was first reported by Sato et al. in 2009 which murine intestinal Lgr5+ stem cells were used to generate organoids with growth factors like WNT, Noggin, R‐spondin, and EGF.^45^ Since then, organoids from various tumor types have been established, including prostate, gastric, pancreatic, and ovarian cancers. Various growth factors and hormones are supplemented in the culture medium to promote self‐renewal and cell differentiation, and their composition is different for different cancers (Table 2).^8^, ^9^, ^46^, ^47^, ^48^, ^49^, ^50^, ^51^, ^52^, ^53^, ^54^, ^55^, ^56^, ^57^

**TABLE 2:** General overview of growth factors in the culture medium of (A) prostate, gastric, spleen, cervical, endometrial and (B) ovarian cancers.

Types of cancer	Prostate	Gastric	Pancreatic	Cervical	Endometrial
(A)					
References	Cheaito et al.^48^	Yan et al.^46^	Loomans et al.^47^	Lõhmussaar et al.^57^	Boretto et al.^144^
Media composition	Advanced DMEM/F12	Advanced DMEM / F12	Advanced DMEM / F12	Advanced DMEM / F12	Advanced DMEM/F12 with L‐glutamine
	HEPES	HEPES		HEPES	HEPES
	Penicillin–Streptomycin	Penicillin–Streptomycin	Penicillin–Streptomycin	Penicillin–Streptomycin	Penicillin–Streptomycin
	Glutamax	WNT3A	B27	Glutamax	Glutamax
	Gentamicin/Amphotericin B	R‐spondin 1	EGF	B27	B27
	Plasmocin	Noggin	N‐acetylcysteine	Nicotinamide	Nicotinamide
	B27	B27	R‐spondin 1	EGF	EGF
	Nicotinamide	N‐acetylcysteine	Gastrin	FGF10	bFGF
	EGF	FGF10		A83‐01	FGF‐10
	FGF2	EGF		N‐acetylcysteine	A83‐01
	FGF10	Gastrin		Noggin	N‐acetylcysteine
	A83‐01	A83‐01		R‐spondin 1	Noggin
	N‐acetylcysteine	Y‐27632		p38 inhibitor	R‐spondin 1
	Noggin			Y‐27632	Insulin Transferrin Selenium
	R‐spondin 1			FGF7	Nitrogen
	p38 inhibitor			Forskolin	p38 inhibitor
	Dihydrotestosterone			CHIR	17‐β Estradiol
	Y‐27632			WNT surrogate	Y‐27632
				17‐β Estradiol	

Types of cancer	Ovarian	Ovarian	Ovarian	Ovarian	Ovarian	Ovarian
(B)						
References	Hill et al.^49^	Kopper et al.^8^	Maru et al.^54^	Hoffmann et al.^52^	Maenhoudt et al.^145^	Nanki et al.^55^
	Wan et al.^9^	de Witte et al.^51^				
	Our group					
Media composition	Advanced DMEM/F12	Advanced DMEM/F12	Advanced DMEM/F12	Advanced DMEM/F12	Advanced DMEM/F12	Advanced DMEM/F12
	HEPES	HEPES	HEPES	HEPES	HEPES	HEPES
	Penicillin–Streptomycin	Penicillin–Streptomycin	Penicillin–Streptomycin	Penicillin–Streptomycin	Penicillin–Streptomycin	Penicillin–Streptomycin
	Glutamax	Glutamax	Glutamax	Glutamax	Glutamax	Glutamax
	B27	Primocin	EGF	B27	B27	B27
	Nicotinamide	B27	Noggin	Nitrogen	Nitrogen	A83‐01
	EGF	Nicotinamide	R‐spondin 1	Nicotinamide	Nicotinamide	N‐acetylcysteine
	FGF2	EGF	Y‐27632	EGF	EGF	Noggin
	FGF10	FGF10		p38 inhibitor	HGF	WNT3A
	A83‐01	A83‐01		Y‐27632	A83‐01	R‐spondin 1
	N‐acetylcysteine	FGF10		BMP2	N‐acetylcysteine	Y‐27632
	Noggin	A83‐01			Noggin	IGF1
	R‐spondin 1	WNT3A			R‐spondin 1	Leu15‐Gastrin I
	p38 inhibitor	R‐spondin 1			p38 inhibitor	
	Hydrocortisone	17‐β Estradiol			17‐β Estradiol	
	Y‐27632	Hydrocortisone			Y‐27632	
		Y‐27632			IGF1	
					NRG1	

Abbreviations: BMP2, bone morphogenetic protein 2; DMEM/12, Dulbecco's modified Eagle medium/nutrient mixture F12; ECF, epidermal growth factor; FGF2, fibroblast growth factor‐2; FGF10, fibroblast growth factor‐10; HGF, hepatocyte growth factor; HEPES, 4‐(2‐hydroxyethyl)‐1‐piperazine‐ethane‐sulfonic acid; IGF1, insulin‐like growth factor 1; NRG1, neuregulin‐1.

**FIGURE 1:**
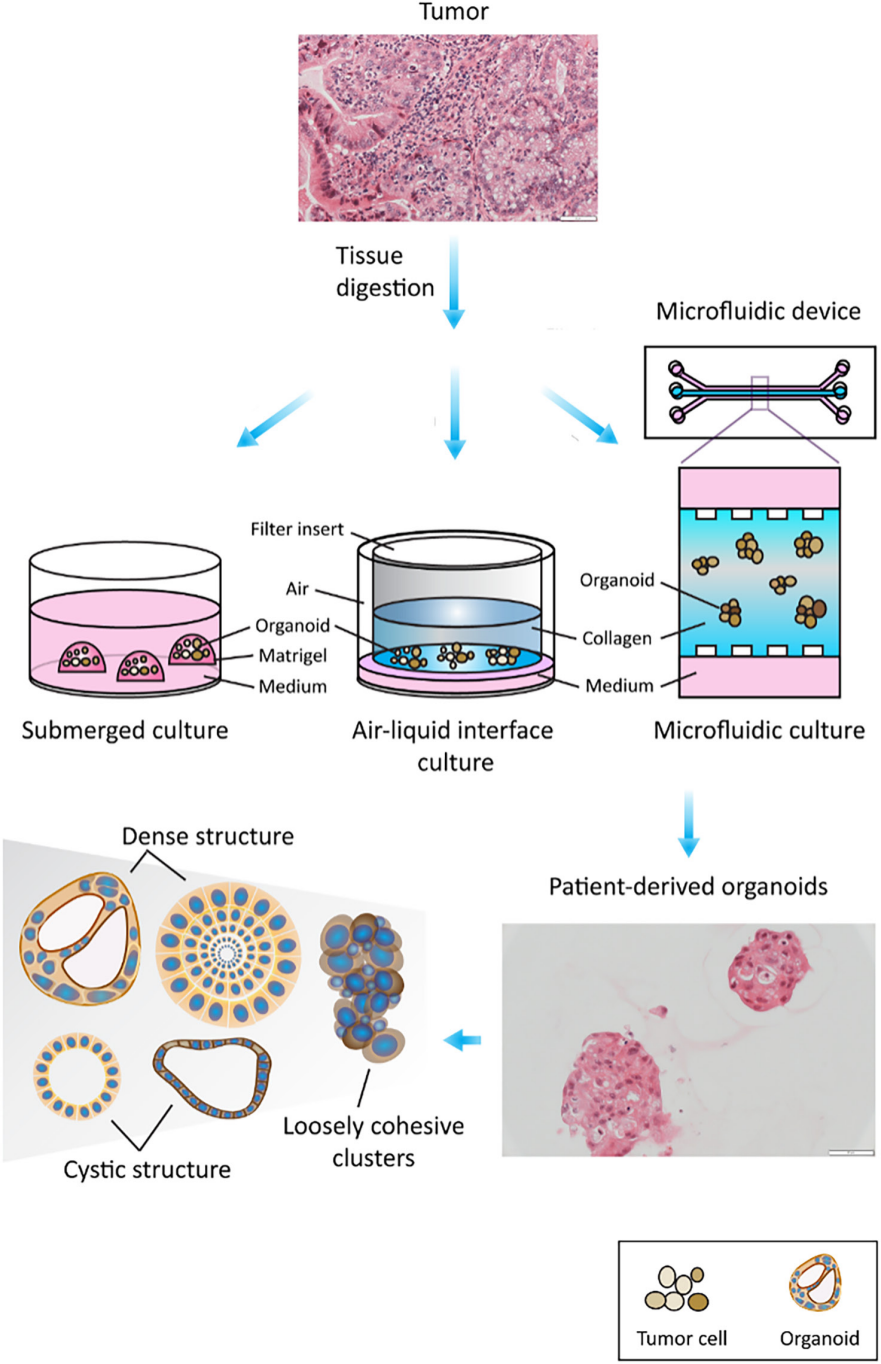
Generation of patient‐derived organoids. The organoid culture system contains digested tumor cells that are embedded either in matrigel in submerged culture, or in collagen in air‐liquid interface culture, or in collagen with medium at both flanks in a microfluidic culture device (Scale bars 50 μM).

There are two main methods of organoid culture (Figure 1). The first method utilizes submerged ECM (see Section 4.2) like matrigel where the tissue is dissociated and the cell suspension is embedded in basement membrane extract within a dome or flat gel covered by enriched culture medium.^14^, ^58^, ^59^ Using this method, immune and other stromal cells may be depleted over time,^45^ which may partly be due to the use of a medium that lacks the growth factors specific to these cells. Matrigel gel also tends to enrich epithelial cells more than other cell populations.^45^ However, a recent article showed that immune cells and stromal cells were retained even in long‐term PDO culture using cryopreserved tumors as detected by single‐cell RNA sequencing (scRNA‐seq).^60^

Another method is microfluidic 3D culture or the air–liquid interface (ALI) culture, in which the native TIME is preserved without the need for subsequent reconstitution of immune or stromal cells.^14^, ^61^ In microfluidic 3D culture, organoids from digested tissues are mixed with collagen forming a spheroid that preserves the TIME components,^14^, ^62^ and are then injected into a microfluidic culture device which often contains a central gel channel flanked by two media channels on both sides.^61^, ^63^, ^64^ Microfluidic chambers contain interconnected multi‐ and microchannels. It provides a dynamic vascular flow in the chamber system, allowing the delivery of chemicals and nutrients to those microfluidic cultures and interaction between different cells such as epithelial cells and stromal cells.^65^ It partially mimics the dynamic metabolic physiology and processes in the body.

In ALI culture, tissues are minced into small fragments thus preserving the architecture and cell diversity of the tumor.^61^ The organoids in collagen gel are grown in an inner transwell dish where its basal surface is in contact with the culture medium in the outside dish, while its apical part is exposed to the air allowing access to the oxygen supply.^14^, ^66^, ^67^ Neal et al. utilized the ALI approach and showed that native CAFs and tumor‐infiltrating T‐cell repertoire could be retained allowing PD1/PDL1 blockade.^67^

Microfluidic and ALI cultures are not as popular as the submerged technique because they require special devices. Researchers need to transfer the organoids from other tissue culture chambers to the microfluidic chambers before starting the experiments. This process will lead to the loss of organoids.^65^ Besides, the microfluidic chambers are not customized for POD culture, and they cannot accommodate organoids larger than 400 μM; otherwise, the organoids may merge inside the chamber.^65^ And as these culture techniques are not very common, the optimization of growth factors is also time‐consuming and challenging.^61^ Lastly, the immune cells may still lose over time in these culture systems and they cannot reflect the recruitment of circulating immune cells into the tumors.^14^

## 4 PATIENT‐DERIVED ORGANOIDS IN EPITHELIAL OVARIAN CANCER

### 4.1 Generation of PDOs in EOC

There has been a substantial increase in the use of PDOs in EOC research in recent years. PDOs from different EOC subtypes have been reported, and the donor tissues can be derived from either the tumors in the ovaries, fallopian tubes, or peritoneum, or metastatic sites like omentum, lymph nodes, bowel, peritoneal or pleural fluid. In general, the tumors are cut mechanically and then digested enzymatically to become single cells. The digested cells are grown in a culture medium supplemented with different growth factors for the establishment of the organoids. Short‐term and long‐term cultures have been reported, and EOC organoids can be expanded up to 30 passages or more than 1 year.^8^, ^49^, ^52^ The overall success rate of PDOs in EOC was 55%–100%.^12^, ^54^, ^64^, ^68^ And this depends on various factors including the use of growth factors and ECM.

#### 4.1.1 Media and growth factors

Some low‐grade tumors like LGSC and borderline ovarian tumor (BOT) can be cultured in media with altered composition from those used to culture normal tissues.^8^, ^54^, ^60^ In high‐grade tumors, the choice of media and growth factors is more complex, and the composition is not standardized and varied in different papers (Table 1). Both commercially available media and self‐prepared media had been used, though there were not too many commercially available media for EOC. Compared to commercially available media, self‐prepared media allows flexibility to tailor the composition of the growth factors that are critical to the formation of the organoids. Using cryopreserved specimens in HGSC, Senkowski et al. demonstrated that the condition of the media affected the growth of organoids and the response to drugs like carboplatin and paclitaxel.^60^

Growth factors such as fibroblast growth factor‐10 (FGF10), neuregulin1 (NRG1), nicotinamide, p38 mitogen‐activated protein kinase inhibitor (SB203580), and transforming growth factor‐beta (TGF‐β) inhibitor (A83‐01 could stimulate the organoid development.^49^, ^53^, ^54^, ^69^ FGF‐4 promotes the tumorigenicity of cancer stem cells, and including FGF‐4 in the medium could enhance organoid formation after passaging.^60^ R‐spondin, bone morphogenetic (BMP) inhibitor Noggin, and epidermal growth factor (EGF) are also required for the stem cells to undergo self‐renewal and differentiation into different cell lineages,^45^ though some groups reported that the use of Noggin and EGF might reduce organoid formation.^60^ Short‐term (2–3 weeks or within 1–2 passages)^49^ and long‐term (4 weeks to 3 months, or more than 6 passages)^52^, ^60^, ^70^ cultures of mouse and human organoids were reported. It is not surprising that the media used are different. Some reported that Rho/ROCK inhibitor (Y‐27632) was essential in the initiation of organoid formation and it could inhibit the growth of organoids derived from healthy fallopian tubes.^49^, ^52^ But this could be omitted in subsequent passages.^49^ Wnt pathway activation was shown to induce growth arrest in HGSC organoids, and so long‐term culture of EOC organoids should be carried out in a low Wnt environment.^52^, ^60^ Several groups also showed that the addition of beta‐estradiol could promote organoid formation and growth over passaging in HGSC.^8^, ^52^, ^53^, ^60^

#### 4.1.2 ECM

ECM is a fibrous network of macromolecules that mainly contains laminin, collagen IV, heparan sulfate proteoglycans, and entactin/nidogen. It has been used to mimic the dynamic nature of TME to provide a scaffolding support to promote organoid formation, and regulate tumor growth and homeostasis through its structural support and associated signaling pathways.^71^, ^72^ In PDOs, a variety of ECM biomaterials have been used to mimic the dynamic nature of TME, including natural hydrogel which can be protein‐based (e.g., Matrigel and Collagen) or polysaccharide‐based (e.g., alginate alginate‐chitosan and agarose), synthetic hydrogel (e.g., polyethylene glycol (PEG), polyglutamic acid (PLGA)and gelatin methacryloyl (GelMA)), decellularized ECM, omental mesothelial models and 3D bioprinting.^73^, ^74^

Matrigel originated from the secretion of Engelbreth–Holm–Swarm mouse sarcoma cells, is the most commonly used scaffold for PDOs.^75^ It is commercially available, has high biocompatibility, and is easy to use.^73^ It mimics basement membrane, and can increase tumor cell stemness and facilitate organoid expansion.^69^, ^76^, ^77^ However, its quality may be affected by batch‐to‐batch variation. There is also a concern about its immunogenic effects on human PDOs due to its murine origin.^78^ In addition, although Matrigel contains more than 1800 ECM proteins, it still may not contain all the elements required for the development of PDOsl.^74^, ^79^

Collagen is the most abundant fibrous protein in mammalian ECM and it can facilitate tumor differentiation, invasion, and metastasis.^80^ Among all types of collagen, collagen‐I is the most common fibrillar type used in cell culture. It is derived from animals like porcine tendon, skin, and bovine lens capsules, and the quality of natural biopolymers may vary from batch to batch.

Polysaccharide hydrogels like alginates and alginate‐chitosan require additional proteins like adhesion peptide Arg‐Gly‐Asp (RGD) which can facilitate cell and promote the organoid forming efficiency and differentiation.^80^, ^81^, ^82^ Besides, their chemical functionality and mechanical properties are stable, because their degradation and remodeling rates are independent of cell‐secreted proteolytic enzymes.^74^, ^80^, ^83^

Synthetic polymeric matrices have low batch‐to‐batch variation.^73^ Some of their mechanical properties, such as stiffness, functionality, and pore size, can be adjustable. However, they have low mechanical strength, and they require biofunctionalization by the addition of cell‐binding peptides to facilitate the growth and differentiation of PDOs.^73^, ^84^, ^85^, ^86^, ^87^, ^88^

Decellularized ECM is isolated from the inhabiting cells. Its quality depends on the donors. However, it has high biocompatibility and can preserve the native ECM and growth factors.^73^ Decellularization from porcine ovary for the creation of a bioengineered ovary had been reported^89^ but the experience in EOC was limited.

Composite hydrogels utilize both proteins and polysaccharides and have an additional benefit on the mechanical properties and adhesion support.^90^ PDOs had been successfully generated from pancreas, intestine, lung, heart, breast, and brain using non‐matrigel methods but the experience in EOC was relatively scarce.^81^, ^85^, ^91^, ^92^, ^93^, ^94^, ^95^, ^96^, ^97^

### 4.2 Characterization of PDOs in EOC

**FIGURE 2:**
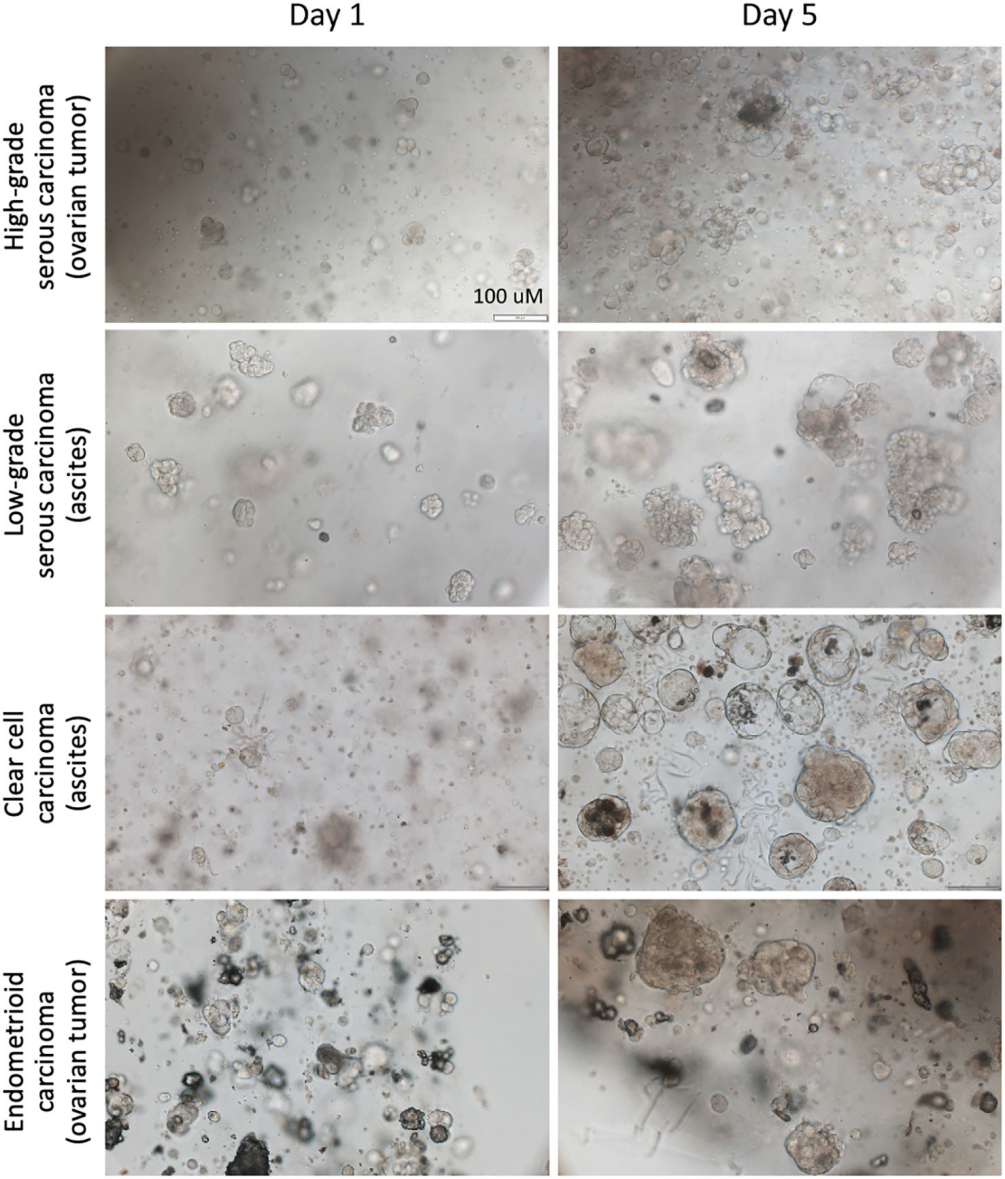
Development of patient‐derived ovarian cancer organoids. PDOs could be derived from different histological subtypes of EOC, and an expansion in size was already evident 5 days (Day 5) after establishment (Day 0) (Scale bars 100 μM).

**FIGURE 3:**
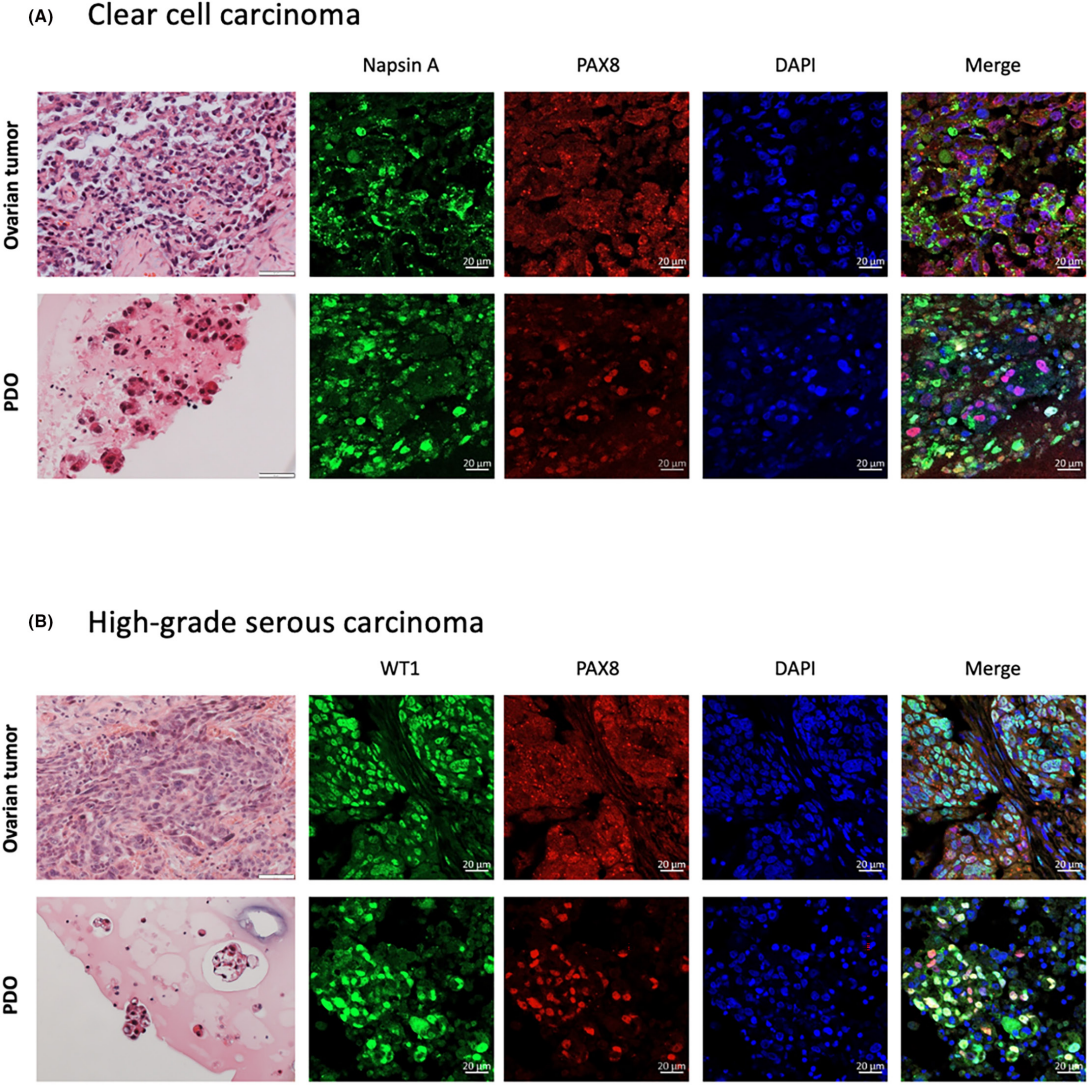
The histological analysis of PDOs using H&E and immunofluorescent staining markers such as (A) Napsin A in clear cell carcinoma and (B) p53 in high‐grade serous carcinoma recapitulated those in the parental tumors. H&E, hematoxylin and eosin; PAX8, paired‐box gene 8 (H&E images, scale bars 50 μM; immunofluorescence images, scale bars 20 μM).

PDOs from different histological subtypes of EOC have been characterized.^8^, ^51^, ^54^, ^55^ Kopper et al. reported 56 long‐term PDOs from 32 patients with HGSC, CCC, LGSC, endometrioid carcinoma (END), serous borderline tumor (SBT), malignant Brenner tumor (MB), and mucinous carcinoma (MC).^8^ After digesting the tumors by Rho/ROCK pathway inhibitor and collagenase, PDOs were generated by submerged approach using Cultrex growth factor reduced Basement Membrane Extract (BME) type 2 (Trevigen, 3533‐010‐02), which is a natural ECM hydrogel that polymerizes at 37°C to form a reconstituted basement membrane consisting of laminin, collagen IV, entactin, and heparan sulfate proteoglycans.^8^ The PDOs were passaged every 1.5–4 weeks for a total of 3–32 times. The PDOs recapitulated the histological features of the original tumors using hematoxylin and eosin (H&E) and immunohistochemical (IHC) staining such as cytokeratin 7, paired‐box gene 8 (PAX8), and p53.^8^, ^49^, ^55^, ^56^, ^98^ In particular, MC, LGSC, END, and CCC organoids formed dense organoids with multiple lumens inside, while BOT organoids had a cystic appearance. Both cystic and dense morphologies with different degrees of circularity and cellular cohesiveness were demonstrated in HGSC organoids.^8^ The development of organoids and the recapitulation of the surface markers using some examples from our group were illustrated in Figures 2 and 3 respectively. The generation and maintenance of these PDOs are described in Supplementary Information.

Whole genome sequencing (WGS)/whole exome sequencing (WES) and genome‐wide CNV analyses of the parental tumors and their PDOs showed that the genomic landscape, including somatic mutations, copy number variation (CNV), single nucleotide variants (SNVs), and structural variants (SVs), can be preserved in the PDOs in both early and late passages.^8^, ^49^, ^51^, ^54^, ^60^ For example, Kopper et al. sequenced 40 PDOs from 22 patients as well as their blood and parental tumors.^8^ WGS showed that the cancer cell content was higher in the PDOs compared to the parental tumors (88.1 ± 23% vs 45.1 ± 9.2%), while CNV and mutation remained similar between the PDOs and the corresponding tumors even after prolonged passaging. Using WES, Hill et al. demonstrated that a median of 98.2% of mutations found in the parental tumors could be retained in the EOC PDOs, and reciprocally, a median of 98.8% of mutations found in the PDOs could also be identified in the parental tumors.^49^ There was no acquisition of new somatic mutations during the short‐term culture for seven to 10 days. Senkowski et al. also showed that the genomic, transcriptomic, and IHC landscapes were reserved in long‐term PDOs after at least 10 passages compared to the tumor tissues.^60^ Although new subpopulations emerged after several months of culture, these cells retained the patient‐specific transcriptional features of the original tumor at single‐cell level. In another report, Hong et al. detected a mixed population of epithelial tumor cells, stromal cells, autologous tumor‐infiltrating lymphoid cells, and tumor‐infiltrating lymphoid cells in colorectal cancer PDOs using flow cytometry and confocal imaging, including CD3+ T cells, macrophage, endothelial cells, CD4+ T cells, CD8+ T cells, NK cells, and monocyte lineage.^62^ These PDOs were sensitive to immunotherapy like anti‐PD1 and anti‐PDL1.

Taken together, PDOs can circumvent some major problems with traditional assays. They can replicate the histological and genomic features in human tumors and can provide a reliable translational platform for cancer research.

## 5 TIPS AND TRICKS FOR PDO GENERATION

Despite the advances in organoid technology, how good organoids can be generated from the parental tumor and how they can be maintained can be a big challenge. The proliferation and survival rate of organoids is affected by several factors, including the quality of the parental quality, the starting cell numbers, the presence of stroma or immune cells, and the supplement of growth factors in the culture medium.

Tissue selection and preparation are the first step of the successful generation of PDOs. It is not unusual for EOC to have necrotic areas when the tumors grow faster than neovascularization. Therefore, tumor tissues should be sampled by experienced clinicians or researchers where necrotic tissues and areas that are covered by mucus should be avoided. It is ideal that the tissues are examined by a pathologist to confirm the tumor content and the quality of the cells. Tumors should be processed in a timely manner, typically within 1–2 h. The tissues should be cut as small as possible. Prolonged enzyme digestion, forceful centrifugation, and prolonged exposure to low temperatures should be avoided. It is also advisable to lyse the red cells with red cell lysis buffer 2–3 times to ensure the clarity of PDOs.

Medium should be freshly prepared with a cocktail of growth factors. In general, the medium should not be kept for over a week. As mentioned above, there is no consensus on the best combination of the growth factors, and cross‐reference to the up‐to‐date literature is needed. R‐spondin, a modulator of the Wnt pathway and a stimulator of adult stem cell proliferation, could either be purchased and added into the basal medium, or prepared using a conditioned medium culturing 293T‐HA‐RspoI‐Fc cell lines in DMEM medium containing 10% fetal bovine serum (FBS) and 1% penicillin–streptomycin (P/S), sequentially with and without zeocin, followed by advanced DMEM with FBS fand P/S.^99^ If the latter is used, it is necessary to ensure that the cell line is authenticated and is free of mycoplasma. If Matrigel is used, it is essential to gently mix it beforehand. Multiple freezes and thaws should be avoided to avoid denaturation of the proteins.

The medium in the PDO culture should be replenished every 2–3 days.^99^ Care must be paid not to aspirate the PDOs. The PDOs should be inspected on a daily basis. When the confluency reaches about 70%, passaging should be considered. Mechanical disruption by gentle pipetting or enzyme splitting using TrypLE can be used. PDOs can be conveniently cryopreserved using commercially available stocking solution. For prolonged culture, it is essential to ensure that the specimens are free of bacterial and mycoplasma contamination.

## 6 APPLICATIONS

PDOs are used to facilitate the understanding of cancer biology by engineering the genomes of the PDOs. The easy generation and perpetuation of PDOs also allow high throughput drug screening and the development of biobanks for future research.

### 6.1 Deciphering cancer biology

PDOs can facilitate the understanding of tumorigenesis. For example, Zhang et al. generated organoids derived from fallopian tube epithelium (FTE) of wild‐type and GEMM mice and then inoculated back to *nu/nu* mice, and showed that certain gene mutations like *TP53* and *Lgr5* could cause HGSC.^100^ They also compared the organoids derived from FTE and ovarian surface epithelium (OSE) using RNA sequencin, and demonstrated that the p53‐signaling pathway was enriched in FTE‐derived tumors, while DNA repair pathways were enriched in OSE‐derived tumors. Similarly, Lõhmussaar et al. created mouse FTE and OSE organoids with knockout of *Trp53*, *BRCA1*, *PTEN*, and *NF1*, either alone or in combination, using CRISPR‐Cas9 to study the tumorigenesis of HGSC, and found that those organoids derived from the oviducts grew faster than those derived from the OSE.^101^

On the other hand, the Wnt/β‐catenin signaling pathway is well known for its role in cancer stemness, tumor progression, and therapeutic resistance.^102^ Hoffmann et al. found that a low‐Wnt environment could upregulate the stemness genes, thus providing a favorable environment for the expansion of HGSC PDOs with or without the knockdown of tumor suppressor genes *p53*, *PTEN*, and *RB*.^52^ Sun et al. explored the mechanisms of cisplatin resistance by RNA sequencing and quantitative PCR of PDOs derived from 4 cisplatin‐sensitive and 6 resistant ovarian cancer tissues.^103^ The authors demonstrated that some genes involved in senescence like *TP53*, *p16*, *and p21*, were suppressed, while some genes involved in glycolysis like *HK2*, *GLUT1*, *and LDHA*, were upregulated in cisplatin‐resistant PDOs compared to sensitive PDOs. Cisplatin‐resistant PDOs also had a higher Aurora‐A immunofluorescent intensity in the resistant PDOs, and further assays suggested that the Aurora‐A/SOX8/FOXK1 signaling pathway was associated with the cisplatin resistance.

PDOs can also help to understand the tumor evolution and heterogeneity. For example, Mo et al. compared paired colorectal (CRC) and liver metastasis (LM) PDOs in patients with colorectal cancer with liver metastasis (CRLM).^104^ It was shown that both CRC and LM PDOs shared the early common driver mutations like *TP53* and *APC* genes. However, some CRC and LM PDOs later accumulated different unique mutation patterns. scRNA‐seq analyses in two patients revealed that there were different proportions of stem‐like cells among CRC and LM PDOs and different patients, highlighting the presence of inter‐ and intra‐patient heterogeneity. Lastly, the morphology of CRC and LM PDOs was different in some patients in their cohort.

### 6.2 Personalized medicine: Drug screening and new drug development

There is a trend of adopting personalized medicine to treat advanced and recurrent cancer patients. Short‐term cultures of PDOs assist the development of personalized medicine in several ways. First, compared to conventional cell lines, PDOs can retain more patients' tumor characteristics and so the results derived from PDOs are more representative and reliable for future clinical use.^9^, ^49^, ^55^, ^56^, ^105^, ^106^ Second, many advanced and recurrent EOC patients present with ascites or pleural effusion requiring tapping for symptomatic relief. The body fluid is easily available and can provide fresh tumor cells for organoid culture. The tapping procedure is relatively non‐invasive and inexpensive. PDOs derived from these samples can provide direct information on the effects after drug treatment using functional assays like adenosine triphosphate (ATP) cell viability assay and transcriptome analysis. Third, the PDOs obtained from different sites at the time of operation can be tested with drugs to study inter‐ and intra‐tumor heterogeneity. In Mo′s article, there was no difference in the responses to 5‐fluorouracil, irinotecan, and oxaliplatin in CRC and LM PDOs in CRLM patients.^104^ Fourth, PDOs can be manipulated genetically to investigate drug response and the underlying mechanisms.^51^, ^55^, ^107^, ^108^ Finally, the drug screening by PDOs can be performed in a high throughput manner using 96‐ or even 384‐well plates. It is less tedious and less expensive compared to PDXs. PDOs have been utilized for drug screening in EOC, such as carboplatin, paclitaxel, gemcitabine, topotecan, poly (ADP‐ribose) polymerase inhibitor, and immunotherapy.^9^, ^49^, ^55^, ^56^, ^68^, ^103^ However, these were limited to small case series without proper sample size calculation. Several clinical trials are ongoing to evaluate the efficacy of PDOs in predicting drug response and survival in EOC (NCT04555473, NCT05290961, NCT05175326, NCT04768270, and NCT05537844).

The most common strategy in personalized medicine is to obtain a fresh tumor biopsy or to use archived tissue samples, extract the DNA, and perform gene profiling by next‐generation sequencing (NGS) to identify if there are any druggable mutations or phenotypes like homologous recombination deficiency (HRD) and tumor mutation burden. However, these tests do not have a functional assay to demonstrate the response. Hill et al. demonstrated that the response to PARPi in patients with EOC might not be accurately predicted by *BRCA* or HR status.^49^ Instead, the functional assays in PDOs like cell viability assay and DNA fiber assay might better correlate with the clinical response to PARPi. Therefore, the different features of NGS and PDOs (Table 3) provide different information and there is a potential to combine both platforms together to predict drug response. Gorski et al. developed PDOs from two patients with neoadjuvant chemotherapy and four chemotherapy‐naïve patients with HGSC.^109^ The PDOs were tested with carboplatin, and the response was correlated with the clinical response.

**TABLE 3:** Potential combination use of PDO culture and NGS in personalized medicine.

	PDOs	NGS
Cell requirement	Fresh	Fresh or archived
	Tumor or body fluid	Usually tumor; cytological specimen may not be feasible if there are enough cells
Sampling procedure	Usually invasive	Usually invasive
Information on druggable mutations	Yes if there is enough DNA	Yes
Direct functional assays for drug screening response	Yes	No
Further mechanistic assays after drug treatment	Yes	No

Abbreviations: NGS, next‐generation sequencing; PDOs, patient‐derived organoids.

## 7 FUTURE PERSPECTIVES

### 7.1 Genetic engineering models

Various genetic engineering methods such as RNAi and CRISPR/Cas system have been used in organoids through electroporation, lipofection, or viral approaches.^110^ Zhang et al. employed CRISPR‐Cas9 techniques to edit multiple genes in organoids derived from mice FTE, and tested the drug response with different gene alterations.^111^ In particular, they found that *Trp53* ^*−/−*^ *;CCNE1* ^*OE*^ *;Akt2* ^*OE*^ *;Kras* ^*OE*^ organoids were more sensitive to gemcitabine than the other models, while *Trp53* ^*−/−*^ *;PTEN* ^*−/−*^ *;Nf1* ^*−/−*^ cells were more sensitive to paclitaxel. These findings were confirmed by mice and human PDO models. Wang et al. also knocked out *FBN1* by CRISPR/Cas9 and lentivirus in EOC, and found that there was an enrichment of the glycolysis and angiogenesis pathways, and cisplatin sensitivity was improved compared to control.^112^

### 7.2 Biobanking

Living organoid biobanks are established by cryo‐preserving PDOs from tumors and normal tissues in liquid nitrogen tank for future research.^113^ They can allow the study of cancer biology in different cell types. Organoid biobanking creates an opportunity for genetic engineering and can help the development of personalized anti‐tumor targeted therapy. Besides, it has been shown that genomic profiles can remain stable in long‐term living organoid culture.^27^ Hence, genomic analyses can be used to compare the cancer biology in PDOs from different patients in the biobanks or to study the tumor evolution of PDOs derived from the same patient at different tumor sites or over different time points. Currently, there is only limited literature on PDOs in cell types other than HGSC.^8^, ^51^, ^53^

### 7.3 Co‐culture models

Co‐culture spheroid models have been used to study immune‐oncology. However, the spheroids are mostly derived from human cancer cell lines that lack other cell populations.^114^, ^115^, ^116^ There is a concern on the compatibility of HLA typing between the immune cells and cancer cell lines. Besides, the use of FBS may potentially interfere with the immune response in the co‐culture spheroid models. PDOs contain different cell lineages including tumor cells and their stem cells, immune cells, and mesenchymal and stromal cells like fibroblasts, which can better resemble the TME and appear more relevant in evaluating immunotherapy compared to co‐culture spheroid models. However, these cells will inadvertently be lost after several rounds of organoid passaging, leaving only the epithelial layers behind.^69^, ^117^ Therefore, co‐culture systems of PDOs and patient‐matched isolated immune cells and/or stromal cells are required.

There are two approaches to perform co‐culture systems in PDOs.^118^ The holistic approach retains all the cells including the endogenous immune cells within the PDOs, where the latter are then expanded and activated, allowing short‐term culture and functional studies. For example, Wan et al. generated co‐cultures by enriching the immune cells in the tumor organoids with IL2, which were then incorporated in 15% Matrigel with DMEM and IL2.^9^ Immune checkpoint inhibitors such as anti‐PD‐1 and bispecific anti‐PD‐1/PD‐L1 were added for 96 h, after which further mechanistic studies like flow cytometry and single‐cell RNA sequencing were performed.

In contrast, the reconstitution approach involves the expansion of PDO and exogeneous immune cells individually before coculturing them together. Dijkstra et al. generated co‐cultures of co‐cultures of tumor organoids and autologous peripheral blood lymphocytes, where the tumor organoids were stimulated by IL‐2 for 24 h before co‐culture and the peripheral blood lymphocytes were stimulated weekly by the tumor.^119^ The enriched tumor‐reactive T cells could then be used to kill the efficacy and mechanism of matched tumor organoids. One drawback of PDOs is the lack of tumor stroma where only the progenitor cells of epithelial origin can be retained.^120^ Yet, CAFs can suppress the immune environment.^121^ These can be easily isolated by digesting the tumors by trypsin, which can then be co‐cultured with the matched PDOs with and without immune cells to study tumor‐stromal interactions.^122^, ^123^, ^124^ The lack of vasculature limits the growth of the PDOs causing necrosis of their center. There have been reports of co‐culturing the PDOs with human umbilical vein endothelial cells with tumor spheroids and fibroblasts,^125^ and engrafting organoids into animals' tissues.^126^, ^127^, ^128^ Vascularized PDOs could also be generated by inducing pluripotent stem cells (iPSCs) to mesodermal progenitor cells (MPCs) using a mesodermal induction medium, the latter of which is then co‐cultured with organoids.^129^

Recently, Malacrida et al. described the generation of tri‐ and tetra‐culture of HGSC, where mesothelial cells, fibroblasts, and adipocytes were isolated from the patient's omentum and were then cultured with the tumor cells in adipocyte gel with or without collagen gel.^130^ This model could allow experiments for up to 21–28 days. The authors used this model to elicit the interaction between platelet and mesothelial cells in tumor invasion.^131^

### 7.4 Other novel methods

There are ongoing developments in tissue culturing techniques to overcome some of the shortcomings of the current PDO cultures. Microfluidics‐based tumor‐on‐a‐chip (TOC) model uses a fabricated chip made of glass, plastic, or polymers with hollow microchannels lined by living cells or tissues cultured under dynamic fluid flow.^132^ This system can be tissue‐based which contains only one channel lined by one type of cells, or organ‐based which contains multiple cell types separated by either porous ECM‐coated membrane or ECM gel.^133^ This novel device allows cell‐to‐cell and even tissue‐to‐tissue interaction and can mimic certain physiological features such as fluid shear stress and drug delivery through dynamic perfusion of culture media or even blood.^133^

New scaffolding materials like recombinant silk microfiber have been used to generate mature human brain organoids, which could facilitate the delivery of oxygen and nutrients, promote self‐assembly, and reduce heterogeneity of cellular organization compared to organoids without silk scaffold.^134^ The use of native ECM by decellularization from native tumor and liver scaffolds had been reported in cholangiocarcinoma, which had a better resemblance to the original tumor TME than ordinary BME.^135^, ^136^

Dynamic cell culture like magnetic levitation is an alternative way to create organoids, where cells are suspended with magnetic beads and organoids are generated under magnetic field.^137^, ^138^ It was postulated that the cell aggregates could be more homogeneous, and the rotating magnetic field may also mimic the mechanical forces of blood flow.^116^ Rotary cell culture systems like spinner bioreactor, rotational bioreactor, and vibrating bioreactor have been used in various cancers.^139^ This method can evaluate the effects of fluid flow on cell behaviors, such as the shear stress from the flow of ascites.^73^ The dynamic movement of cell culture by rotation could also reduce cellular adhesion and increase the supply of nutrients and oxygen to the organoids.^139^, ^140^

3D bioprinting techniques use cell‐compatible bioinks and post‐printing crosslinking methods to build 3D models.^75^, ^141^ Examples include extrusion‐based technique where 3D models are constructed layers by layers using gelatin‐methacryloyl,^142^ and droplet‐based technique which involves fabrication of microtissue cultures and formation of 3D models by drops in hydrogels that can control spatial organization and density of cells.^143^ However, these techniques are costly and the experience is still limited.

## 8 CONCLUSION

PDOs have emerged as a valuable and powerful tool that can advance the knowledge in cancer biology and assess drug response. As a more complex system that at least partially retains the architecture and cell components of the tumor, this model can better reflect the tumor biology compared to conventional 2D systems. PDOs are now commonly incorporated in EOC research as well. However, there are still limitations in the current models. The choice of the culture medium, growth factors, and ECM are not standardized, and the quality of these reagents may vary from batch to batch. Another major pitfall is the loss of the components in TME after several passages of PDOs. Next‐generation organoids like co‐culture models incorporate different cells like immune cells and CAF may partially ameliorate this problem. However, the protocols are not standardized and the success rates vary. Gene‐engineered organoids using different techniques like CRISPR‐Cas9 have been developed. These models can help researchers further understand the etiology of EOC and drug resistance mechanisms. While there is a potential to employ PDOs in personalized medicine, further research is needed to refine these techniques and identify the best models before these can be translated into clinical care.

## AUTHOR CONTRIBUTIONS

**Wai Sun Chan:** Methodology (equal); visualization (lead); writing – original draft (equal); writing – review and editing (equal). **Xuetang Mo:** Methodology (equal); visualization (supporting); writing – original draft (equal); writing – review and editing (equal). **Philip Pun Ching Ip:** Methodology (equal); visualization (equal); writing – review and editing (equal). **Ka Yu Tse:** Conceptualization (lead); supervision (lead); writing – original draft (equal); writing – review and editing (lead).

## FUNDING INFORMATION

None.

## Supporting information


Supplementary Information

## Data Availability

Data sharing is not applicable to this article as no new data were created or analyzed in this study.
