# Supplementary material for: Patient‐derived organoid culture in epithelial ovarian cancers—Techniques, applications, and future perspectives
Source: Cancer Med. 2023 Sep 30;12(19):19714–31. doi: 10.1002/cam4.6521 (PMC10587945; doi:10.1002/cam4.6521)
Supplement: Supplementary file 1 — Supplementary Information [file CAM4-12-19714-s001.docx]

Supplementary Information

The generation and maintenance methodology used by our group was modified by the methods described by Hill et al.^49^ and is described below:

Tissue Preparation and Organoid Culture.

Tumor tissues were placed in advanced DMEM/F12 medium in a clean bottle after collection and were processed within 2 h. Tumor tissues were diced into 2 mm using knives and scissors and were then shaken in advanced DMEM/F12 with 2.5 mg/mL type II Collagenase at 37°C for about 20 min. The homogenate was diluted with advanced DMEM/F12 and passed through a 70 µM filter. The follow-through was then centrifuged at 300 g for 5 min. The pellet was washed with red cell lysis buffer and then advanced DMEM/F12 again. The cell pellet was then resuspended with 80% Matrigel on ice. About 3 to 4 drops of Matrigel were seeded on each well of a pre-warmed 6-well low-attachment plate. The plate was incubated at 37°C for 15–20 min. A culture medium with growth factors described in Table 1B was then added. The culture medium was replenished every 3 days.

Organoid Subculture.

The organoids were split when they became confluent. 1 mL of cool cell harvesting solution was added to each well to dissolve the Matrigel. The organoids were pipetted up and down and were left at 4°C for 30 min. It was then centrifuged at 300 g at 4°C for 5 min. The pellet was resuspended with 1 mL of prewarmed TrypLE and was incubated at 37°C for 3 min. Medium was added and the suspension was centrifuged at 300 g at 4°C for 5 min. After removing the supernatant, the cells were resuspended with Matrigel again and were split into 1:2 or 1:3.

Organoid Live Imaging.

The growth of the organoids was checked under a light microscope. Pictures in Figures 2 and 3 were taken by Olympus DP74 Color CMOS brightfield imaging.

Cell Block Preparation.

The medium was aspirated. Organoids were centrifuged with 1x PBS twice at 400 g at 4°C for 3 min. Cell harvesting solution was added and the mixture was kept at 4°C for 60 min. It was washed away with 1x PBS and then incubated with 4% PFA for at least 1 h. After removing the PFA, the organoid pellet was resuspended with pre-heated Histogel, which was then placed at 4°C for at least an hour to allow it to solidify. The cell blocks were made.
